# MiR‐24 enhances radiosensitivity in nasopharyngeal carcinoma by targeting SP1

**DOI:** 10.1002/cam4.660

**Published:** 2016-02-29

**Authors:** Min Kang, Jingjian Xiao, Jun Wang, Pingting Zhou, Tingting Wei, Tingting Zhao, Rensheng Wang

**Affiliations:** ^1^ Department of Radiation OncologyThe First Affiliated Hospital of Guangxi Medical UniversityNanning530021GuangxiP.R. China

**Keywords:** miR‐24, nasopharyngeal carcinoma, radiosensitivity, SP1

## Abstract

Radioresistance remains a major problem in the treatment of patients suffering from nasopharyngeal carcinoma (NPC). A better understanding of the mechanisms of radioresistance may generate new strategies to improve NPC patients' responses to therapy. This study was designed to investigate the effect of microRNA on the radiosensitivity of NPC cells. A microRNA microarray indicated that miR‐24 was downregulated in NPC cell lines and tissues. Furthermore, cell proliferation was suppressed and radiosensitivity increased when miR‐24 was ectopically expressed in NPC cells. Specificity protein 1 (SP1) was additionally verified as a direct functional target of miR‐24, which was found to be involved in cell viability as well as the radiosensitivity of NPC cells. In conclusion, the results of this study suggest that the miR‐24/SP1 pathway contributed to the reduction in radioresistance in human NPC and that it may thus represent a therapeutic target.

## Introduction

Nasopharyngeal carcinoma (NPC) is a type of cancer derived from epithelial cells located in the nasopharynx 1. The incidence of NPC is higher in southern China and southeast Asia than in western countries 2. The current primary therapeutic strategy for patients with NPC is radiotherapy 3. Nevertheless, radioresistance remains one of the serious obstacles to successful treatment, and can result in distant metastases as well as local recurrence in some patients with NPC after radiation treatment. Therefore, particular emphasis has been accorded to finding effective radiosensitizers as well as to improving the effectiveness of NPC therapy as well as patient survival rates.

MicroRNAs (miRNAs) are one type of small noncoding RNAs (20–24 nucleotides) which posttranscriptionally modulates gene expression through negative regulation of the stability or translational efficiency of their target mRNAs 4, 5. These effects are obtained by miRNA binding to the 3′‐untranslated region (3′UTR) of their target mRNAs, which can reduce the expression of the associated protein. Some reports have shown that miRNAs can regulate the expression of different kinds of genes involved in embryonic development and human disease 6, 7, 8, especially in cancer 9, 10. Furthermore, a number of studies have shown that miRNAs can function as either oncogenes or tumor suppressors to regulate all kinds of basic cellular functions, including proliferation, migration, differentiation, apoptosis, cell cycle, and angiogenesis, and therefore induce tumorigenesis 11, 12. Notably, some recent studies have indicated a relationship between the expression of certain miRNAs, including miR‐29c and miR‐608, and the success of radiotherapy treatment, especially in NPC 13, 14.

Of the above mentioned miRNAs, miR‐24 is abundant, conserved between species, and expressed in normal tissues, such as adipose, kidney, mammary gland, and differentiated skeletal muscle tissues 15. In addition, miR‐24 is encoded by the corresponding gene that maps to the human chromosome 19p13 and 9q22 regions. It has been reported that miR‐24 is a tumor suppressor in a variety of human cancers, including tongue squamous cell carcinoma, bladder cancer, osteosarcoma, and gastric cancer 16, 17, 18, 19. It is also implicated in multiple malignancy‐related processes, including cell proliferation, apoptosis, invasion, and metastasis 20, 21, 22, 23. It has also been reported that miRNAs are involved in several signaling pathways and DNA damage repair processes, and that it can affect cellular radiosensitivity 24. However, to our knowledge, there has been no previous research focused on the role of miR‐24 in response to irradiating nasopharyngeal carcinoma.

The aim of this study was to elucidate the role of miR‐24 in NPC and to investigate the functions of its ectopic expression in NPC cell proliferation and radiosensitivity, which should indicate that miR‐24 is involved in the initiation and progression of NPC. Additionally, we found that specificity protein 1 (SP1) is a direct target gene that mediates the oncogenic effect of miR‐24 in NPC. Thus, these findings provide valuable clues toward understanding the molecular mechanisms that regulate the pathogenesis of NPC and may be helpful in raising NPC cell radiosensitivity and improve the treatment of NPC in the future.

## Materials and Methods

### Cell culture and tissue samples

The adherents CNE1,CNE2, and TWO3 were cultured at a density of 1 × 10^5^ cells in DMEM medium containing 10% FBS, 100 IU/mL penicillin, and 100 mg/mL streptomycin. Cell C666‐1 was cultured at a density of 1 × 10^5^ cells in IMDM medium containing 15% FBS, 100 IU/mL penicillin, and 100 mg/mL streptomycin. The cells were cultured in a CO_2_ incubator at 37°C, in conditions of 90% humidity and 5% CO_2_, as previously described 25. They were passaged via trypsinization (trypsin‐EDTA, Gibco BRL Co. Ltd. Gaithersburg, USA) 3 times every week.

Both tumor and nontumor samples were confirmed as such by pathological examination. No patients had a medical history of other malignant tumors, radiotherapy or chemotherapy; their clinical and pathological data are displayed in Table 3. There were 55 male and 27 female in the cohort of 82 nasopharyngeal carcinoma patients, with an average age of 46.3 (ages ranged from 22 to 81).Their clinical stages were defined according to the 2002 AJCC/UICC staging classifications. Of the 82 cases, six were stage I; 23 stage II, 30 stage III, and 23 stage IV. There were 60 cases of metastatic malignant tumors in cervical lymph nodes and 22 cases of nonmetastatic malignant tumors. Twenty‐two nasopharyngeal chronic inflammation tissue samples were randomly collected from 13 male and 9 female subjects (average age 43.7, ages ranged from 24 to 67) as control. Collection and use of tissue samples were approved by the human research ethics committee of the First Affiliated Hospital of Guangxi Medical University.

### Screening and identification of microRNAs by microRNA microarray

Screening and identification of microRNAs was conducted by microRNA microarray according to the manufacturer's protocols. After extraction of total RNA, the microRNAs were extracted and marked. Hybridization with microRNA chips was conducted on the concentrated marker sample. Fluorescence intensity imaging of microRNAs and data analysis was then performed, using relative values as expression intensity.

### RNA extraction, reverse transcription, and quantitative RT‐PCR

Total RNA was extracted using TRIzol reagent (Life Technologies, Grand Island, NY) and reverse transcribed using Bulge‐Loop^TM^ microRNA specific RTprimers (RiboBio Co., Guangzhou, China) and M‐MLV reverse transcriptase (Promega, Madison, WI) to quantify the expression of miR‐24, and random primers (Promega) and M‐MLV reverse transcriptase (Promega) were used to quantify the expression of SP1 mRNA. The following PCR primers were used for SP1 and glyceraldehyde‐3‐phosphate dehydrogenase (GAPDH): SP1 forward, 5′‐GGAUGGUUCUGGUCAAAUATT‐3′; SP1 reverse, 5′‐UAUUUGACCAGAACCAUCCTT‐3′; GAPDH forward, 5′‐CTCCTCCTGTTCGACAGTCAGC‐3′ and GAPDH reverse, 5′‐CCCAATACGACCAAATCCGTT‐3′. Quantitative RT‐PCR reactions were performed in a CFX96 TouchTM sequence detection system (Bio‐Rad, Hercules, CA) using Platinum SYBR Green qPCR SuperMix‐UDG reagents (Invitrogen, Carlsbad, CA). RNU6B (U6) and GAPDH were used as controls for normalization, while relative expression levels were calculated using the 2^−ΔΔCT^ method. All experiments were performed in triplicate.

### Establishment and transfection of stable cell lines

In this study, primary cells from passage 4–10 were used for the establishment of stable cell lines. Regarding transfection, when cells reached 90% confluency, they were transfected with miRNA plasmids using Lipofectamine 2000 (Life Technologies, Grand Island, NY) and incubating with OptiMem‐I media for 4 h. Then, the cells were transferred into fresh Dulbecco's Modified Eagle Medium, which contains 10% fetal bovine serum. In order to establish stable cell lines with miRNA overexpression, 2 days after transfection, 1 mg/mL G418 (Invitrogen, Carlsbad, CA) was added to the medium. The transfected cells expressing neomycin‐resistant genes survived in the selective medium. By using conventional cloning techniques for expansion, after 2 weeks the cells were picked individually. Part of the stable cell lines was identified by verifying the expression of miRNA using quantitative RT‐PCR, as previously described 26.

### Clonogenic survival assays

For clonogenic survival assays, CNE‐1 and CNE‐2 cells were seeded into six‐well plates at specific cell densities in triplicate, after 48 h of transfection of miR‐24 mimic or SP1 siRNA, followed by exposure to the indicated doses of radiation (0, 2, 4, 6, 8, or 10 Gy) by using 6 MV X‐rays generated by linear accelerators (Varian 2300EX; Varian, Palo Alto, CA) at a dose rate of 3 Gy/min. Following 10–16 days of incubation at 37°C, the cells were fixed using 100% methanol and stained using 1% crystal violet (Sigma ‐ Aldrich Co., St. Louis, MO, USA). Microscopic inspection (Olympus IX71; Olympus, Tokyo, Japan) was used for the colonies containing ≥50 normal‐looking cells. As described previously 20, the surviving fraction was calculated. The multitarget single‐hit model was fitted to the data to generate survival curves using the formula: SF = 1‐(1‐e‐D/D0)N. The sensitization enhancement ratio with a survival fraction of 10% (SER10) was calculated later. Each experiment was independently performed at least three times.

### MTT assay

For the MTT assay, transfected CNE‐1 or CNE‐2 cells were seeded into 96‐well plates at 1000 cells per well and cell viability was examined at different time points (1, 2, 3, 4, and 5 days after seeding). Briefly, the cells were stained with 20 *μ*L of MTT dye (0.5 mg/mL; Sigma) for 4 h, the media were removed, 100 *μ*L of dimethyl sulfoxide (Sigma) was added, and absorbance was measured at 490 nm using a spectrophotometric plate reader.

### Tumor xenograft and irradiation therapy

For tumor transplantation, CNE1 cells were transfected with or without miR‐24 mimic and then suspended in 100 *μ*L PBS and mixed with 20% matrigel. Tumors were generated by prepared subcutaneously injecting 2 × 10^6^ cells/mL cells into the abdomen of BALB/C mice. Seven days after the cells were injected, the mice were divided into two groups: a control group and a radiotherapy group. For radiotherapy, tumor inoculation sites received a single dose of 12 Gy/1f/1d. In brief, mice in the control group and the radiation therapy group were anesthetized by intraperitoneal injection with 10% chloral hydrate (350 mg/kg). The four limbs were fixed with medical proof fabric. The body surface areas of the tumor locations were marked with a marker pen and compensated with 1 cm petrolatum gauze. The target areas were vertically irradiated with X‐ray from a 6MV‐X (Elekta Limited, West Sussex, UK), with under conditions of 100 cm source distance from the target area skin and 12 Gy/1f/1d. The irradiated field was 2.0 cm × 2.0 cm. During radiotherapy, mice were kept in a waking state. During therapy, tumor volumes were measured every 2 days using calipers as length × width × depth. Sixteen days after inoculation, animals were killed and tumors were removed for quantitative real‐time PCR and western blot assay.

### Western blotting analysis

For western blotting analysis, as previously described 27, the cells were washed, harvested, and prepared. Using 8% SDS‐PAGE, a total of 30 mg protein was separated and then transferred onto a nitrocellulose membrane. The membranes were then incubated in primary antibodies for SPp1 (1:1000) with 5% nonfat milk in Tris‐buffered saline and Tween‐20 (0.1%) for 1 h at room temperature and then actin antibody (1:5000) was added and the mixture left for a further hour. After washing three times, the resulting mixture was exposed to a goat anti‐rabbit or anti‐mouse secondary antibody conjugated with horseradish peroxidase. The signals of the immunoreactive bands were identified by electrochemiluminescent detection.

### Luciferase reporter assay

The 3'‐UTR segments of SP1 mRNA containing miR‐24 binding sites were amplified by PCR from human genomic DNA, and then inserted into the Xba1‐site of the pGL3 vector (Promega), here called pGL3‐SP1‐wt. By using QuikChange site‐directed mutagenesis kit (Stratagene, La Jolla, CA) mutations were introduced into the predicted binding sites of pGL3‐SP1‐wt, and the mutant vector was named pGL3‐SP1‐mut. CNE1 cells seeded in 24‐well plates were transfected with wild‐type or mutant reporter plasmid vector by Lipofectamine 2000. After the second transfection, the cells were transfected once more with miR‐24 inhibitor or negative control for 6 h. After 36 h, the dual luciferase assay system (Promega) was used for the measurement of luciferase activity. The firefly luciferase activity of each sample was normalized to Renilla luciferase activity.

### Immunohistochemical staining

The expression of SP1 in the sections from the paraffin‐embedded subcutaneous cancer tissue was assessed by immunohistochemical staining, as described previously 28. Rabbit polyclonal anti‐SP1 antibody (1:100; Proteintech, Chicago, IL) was used.

### Statistical analyses

Spearman's rank correlation test was used for correlation analysis between the predicted target gene protein levels and the endogenous miR‐24 levels measured previously by RT‐PCR. Pearson's Chi‐Square tests were used to compare target gene expression levels to clinicopathological characteristics. All analyses were performed using SPSS 16.0 for Windows (SPSS Inc., Chicago, IL). All tests were two‐tailed, and *P *<* *0.05 was considered statistically significant.

## Results

### microRNA chip technology was used to detect the expression of miRNA in nasopharyngeal carcinoma tissues and normal tissues

Total RNA was extracted from nasopharyngeal carcinoma and normal tissues. Using a spectrometer for quantification, denatured gel electrophoresis with formaldehyde was employed to test the quality of all RNA 29, 30 snd to analyze the microRNA (miRNA) expression profile in nasopharyngeal carcinoma and normal tissues. As shown in Tables 1 and 2, there were 30 downregulated microRNAs (miR‐24, miR‐229, etc.) and 16 upregulated microRNAs (miR‐27c, miR‐145, and miR‐162, etc).The downregulation of miR‐24 was one of the most evident results.

**Table 1:** Downregulated microRNAs

miRNA	Fold change	*P* value
hsa‐miR‐288	−5.87	0.0006
hsa‐miR‐27a	−4.75	0.0183
hsa‐miR‐27b	−3.92	0.0249
**hsa‐miR‐24**	−**3.83**	**0.0032**
hsa‐let‐7	−3.29	0.0058
hsa‐miR‐17‐3p	−2.87	0.0052
hsa‐miR‐143	−2.78	0.0380
hsa‐miR‐159	−2.65	0.0376
hsa‐miR‐104	−2.19	0.0287
hsa‐miR‐181b	−1.73	0.0280
hsa‐miR‐19a	−1.65	0.0177
hsa‐miR‐29c	−1.23	0.0143
hsa‐miR‐229	−1.14	0.0115

**Table 2:** Upregulated microRNAs

miRNA	Fold change	*P* value
hsa‐miR‐27c	1.43	0.0037
hsa‐miR‐291a‐5p	1.46	0.0065
hsa‐miR‐145	1.71	0.0453
hsa‐miR‐162	1.76	0.0032
hsa‐let‐7 g	1.83	0.0251
hsa‐miR‐29a	2.27	0.0081
hsa‐miR‐189	2.71	0.0427
hsa‐miR‐146b	3.07	0.0376
hsa‐miR‐104	3.45	0.0287
hsa‐miR‐225	3.84	0.0280

### miR‐24 suppression in tissues and cell lines

**Figure 1:**
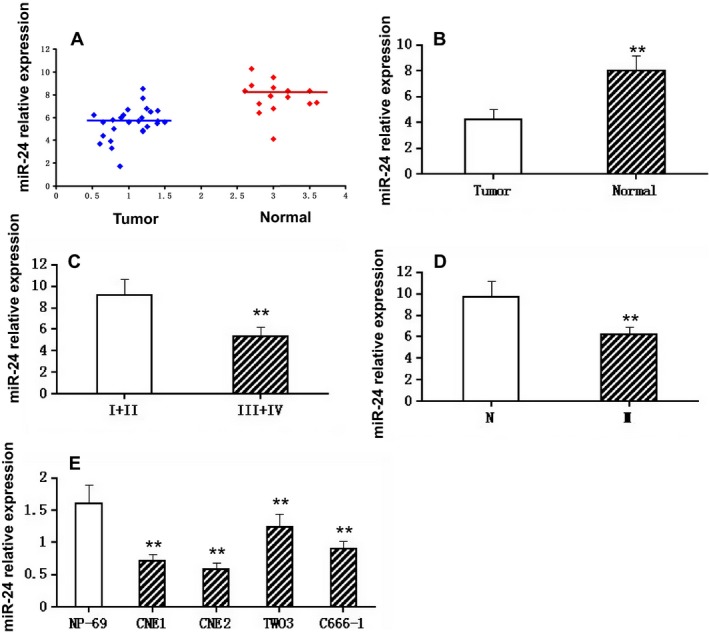
Expression of miR‐24 in various tissues or cell lines. (A). Relative expression of miR‐24 in non‐tumor tissues (*n* = 12) and NPC tissues (*n* = 42). RT‐PCR was used to determine the expression of miR‐24, which was normalized to U6 expression to obtain relative expression. N, non‐tumor tissues; T, tumor tissues. (B). Histograms of the average relative expression of miR‐24 in non‐tumor tissues (*n* = 12) and NPC tissues (*n* = 42). Data are means ±SD. Asterisks indicate values that are significantly different from the values of non‐tumor tissues (*P* < 0.01). (C). Histograms of the average relative expression of miR‐24 in patients at stages I+II and III+IV. Data are means ±SD. Asterisks indicate values that are significantly different from the values of patients at stage I+II (*P* < 0.01). (D). Histograms of the average relative expression of miR‐24 in patients without and with lymph node metastasis. Data are means ±SD. Asterisks indicate values that are significantly different from the values of patients without lymph node metastasis (*P* < 0.01). M, lymph node metastasis. (E). Histograms of the average relative expression of miR‐24 in normal nasopharyngeal epithelial cell line (NP‐69) and NPC cell lines (CNE1, CNE2, TWO3, and C666‐1). Data are means ±SD. Asterisks indicate values that are significantly different from the values of NP‐69 (*P* < 0.01).

RT‐PCR was used for the analysis of the expression of miR‐24. Our data clearly showed that the expression of miR‐24 in the tissues of NPC patients was lower than in the tissues of control subjects (*P *<* *0.01) (Fig. 1A and B). As shown in Table 3, the expression of miR‐24 was lower, but it was higher in the T stage, N stage, and clinical stage (*P *<* *0.01). Notably, compared with the growth in the clinical tumor‐node‐metastasis stage (Fig. 1C), as well as in the lymph node metastasis (Fig. 1D), the expression of miR‐24 was instead lower. The rates of non‐CR in miR‐24 low‐ and high‐expression groups were 79.2% versus 20.8%, respectively (*P *<* *0.001), which indicated that low expression of miR‐24 can mark a poor prognosis in patients. Furthermore, the expression of miR‐24 in NPC cell lines CNE1, CNE2, TWO3, and C666‐1 was lower than that in the normal nasopharyngeal epithelial cell line NP‐69 (*P *<* *0.01, Fig. 1E). In sum, the expression of miR‐24 in NPC patients' tissues and cell lines was lower.

**Table 3:** Clinicopathologic characteristics in nasopharyngeal carcinoma patients

	miR‐24 expression level			
	All cases	Low expression	High expression	*P* value
Age				
≥50 year^a^	44	28 (63.6%)	16 (36.4%)	
<50 year	38	21 (55.3%)	17 (44.7%)	0.182
Gender				
Female	27	16 (59.3%)	11 (40.7%)	
Male	55	32 (58.2%)	23 (41.8%)	0.842
T classification				
1	12	4 (33.3%)	8 (66.7%)	
2	28	11 (39.3%)	17 (60.7%)	
3	26	15 (57.7%)	11 (42.3%)	
4	16	11 (68.8%)	5 (31.2%)	0.001
N classification				
0	22	9 (40.9%)	13 (59.1%)	
1	31	15 (48.4%)	16 (51.6%)	
2	22	14 (63.6%)	8 (36.4%)	
3	7	5 (71.4%)	2 (28.6%)	0.006
Clinical stage				
I	6	2 (33.3%)	4 (66.7%)	
II	23	10 (43.5%)	13 (56.5%)	
III	30	19 (63.3%)	11 (36.7%)	
IV	23	17 (73.9%)	6 (26.1%)	0.001
Therapy response				
CR	58	30 (51.7%)	28 (48.3%)	
Non‐CR	24	19 (79.2%)	5 (20.8%)	<0.001

WHO, World Health Organization; CR, complete response; Non‐CR(including PR, partial response; NC, no change; PD, progressive disease).

a Mean age.

### miR‐24 suppresses NPC cell viability and sensitizes NPC cells to radiation

**Figure 2:**
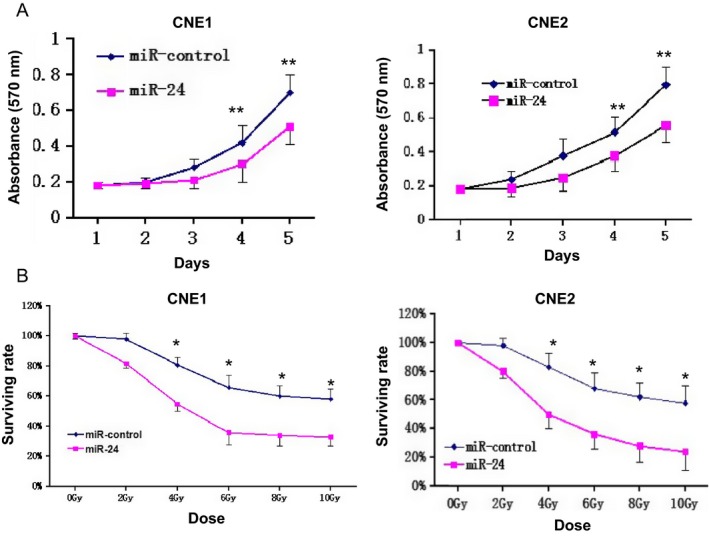
miR‐24 suppresses NPC cell viability and sensitizes NPC cells to IR. (A). Cell viability was determined following transfection with miR‐24 mimic or miRNA mimic NC at day 1–5 in CNE‐1 and CNE‐2 cells(**P < 0.05). (B). Clonogenic survival assays of CNE‐1 and CNE‐2 cells treated with miR‐24 mimic or miRNA mimic NC followed by various doses of irradiation. Surviving fractions were calculated as described. SER10, sensitizer enhancement ratio at 10% survival (*P < 0.01).

Correlation analysis, including a clonogenic survival assay and an MTT assay, and then transfection of CNE‐1 and CNE‐2 cells with miR‐24 mimic or negative control, was conducted to evaluate the effects of miR‐24 overexpression on cell viability as well as the radiosensitivity of NPC cells. Compared with the controls, the spread of NPC cells was clearly reduced by the exceptional expression of miR‐24 (*P *<* *0.05, Fig. 2A). Moreover, based on the differential doses of radiation, the survival rates of cells transfected with miR‐24 mimics was distinctly reduced while those of cells transfected with negative controls was relative higher (*P *<* *0.01, Fig. 2B).

### SP1 is a direct target of miR‐24, and is involved in NPC cell radiosensitivity and growth

**Figure 3:**
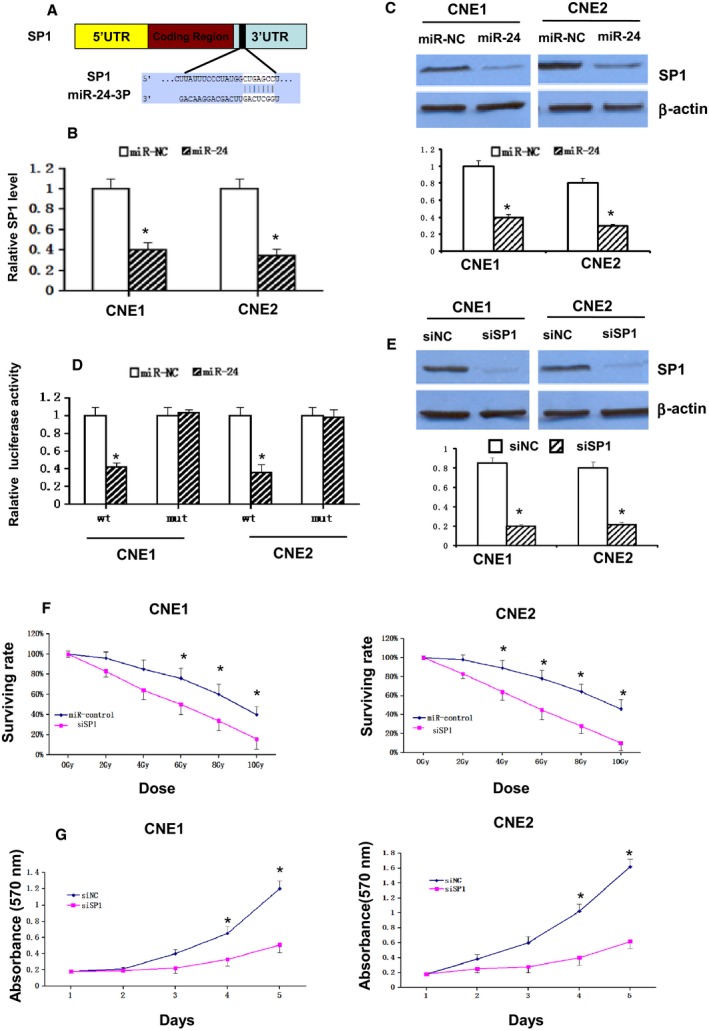
SP1 is a direct target of miR‐24 and involved in NPC cell radioresistance and growth. (A). Predicted miR‐24‐3p binding sequences in the 3′UTR of SP1. Quantification of (B). SP1 mRNA expression and (C). protein levels following transfection with miR‐24 mimic or miRNA mimic NC. (D). CNE‐1 and CNE‐2 cells were co‐transfected with a wt or mut SP1 3′UTR reporter gene and a miR‐24 mimic or a miRNA mimic NC. Wt and mut miR‐24 target sequences of the SP1 3′UTR are indicated. (E). Western blot analysis for SP1 48 h following transfection with SP1 siRNA or a negative control. (F). Clonogenic survival assays of CNE‐1 and CNE‐2 cells treated with SP1 siRNA or an NC followed by various doses of radiation. Surviving fractions were calculated as described. (G). Cell viability was determined following transfection with SP1 siRNA or an NC at days 1–5 in CNE‐1 and CNE‐2 cells. Values are presented as the mean ± standard deviation. *P < 0.05 versus miR‐NC. SP1, Specificity protein 1; miR, miRNA; NC, negative control; wt, wild‐type; mut, mutant; siRNA, small interfering RNA; UTR, untranslated region; hsa, *Homo sapiens*.

To investigate the molecular mechanism whereby miR‐24 raises the radiosensitivity of NPC cells, three publicly available databases were used: Target Scan (http://www.targetscan.org), miRanda (http://www.microrna.org/microrna/home.do), and Pictar (http://pictar.mdc-berlin.de/). SP1 was verified as a potential target of miR‐24 (Figs. 3A). Ectopic expression of miR‐24 strongly suppressed the expression of SP1 mRNA and protein (Fig. 3B and C). Subsequently, the luciferase reporter vectors containing wild‐type or mutant miR‐24 target sequences of the SP1 3′UTR were constructed (Fig. 3D, lower panel). To assess whether SP1 is an immediate target of miR‐24, we used a luciferase reporter assay. The overexpression of miR‐24 is certainly capable of significantly suppressing the luciferase activity of the 3′UTR of SP1 when compared with the activity of a futile reporter gene (Fig. 3D, upper panel), an observation that illustrated the specificity of miR‐24 in targeting the SP1 3′UTR. These results indicate that SP1 is an immediate target of miR‐24 in NPC cells. CNE‐1 and CNE‐2 cells were transfected with SP1 siRNA or control siRNA to prove whether or not the miR‐24‐enhanced radiosensitivity is related to the direct targeting of SP1. Conversely, eliminating the expression of SP1 significantly increased the radiosensitivity of CNE‐1 and CNE‐2 cells (Fig. 3E and F). Additionally, silencing the expression of SP1 significantly suppressed the growth of CNE‐1 and CNE‐2 cells, which was demonstrated by the proliferation assay (Fig. 3G). In sum, SP1 targeting by miR‐24 directly increases radiosensitivity,.

### Local radiotherapy suppressed tumor growth in mice bearing nasopharyngeal carcinoma tumor

**Figure 4:**
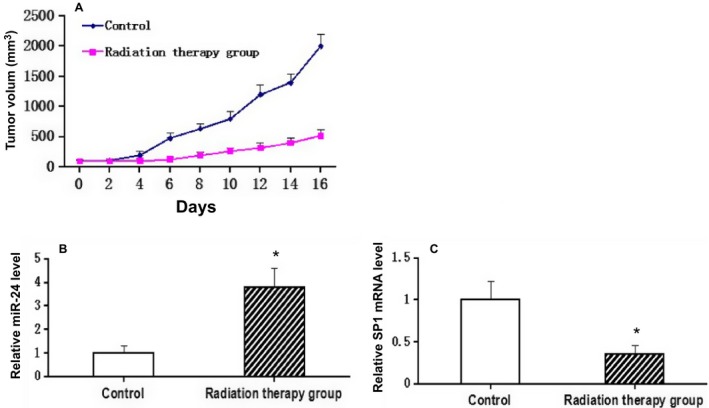
Effect of local high‐dose irradiation on tumor growth and expression of miR‐24 and SP1 in mice bearing nasopharyngeal carcinoma tumor. Seven days after BALB/C mice transfected with CNE1 cells, tumors were irradiated with a single dose of 12 Gy/1f/1d, whereas control mice received no radiotherapy. (A). Growth of tumor in irradiated mice. (B). Expressions of miR‐24 and (C).SP1 were determined by using Quantitative Real‐Time PCR in tumors. The results were represented as mean ±SD. *P < 0.05 compared with control.

Following subcutaneous transplantation, BALB/C mice bearing nasopharyngeal carcinoma tumors were locally irradiated with a single dose of 12 Gy for 7 days, which resulted in a significant reduction in tumor growth (Fig. 4A). When we applied qRT‐PCR analysis on expression of miR‐24 and SP1 in tumors treated with radiation therapy, the results showed an increase in levels of miR‐24 (Fig. 4B) and a decrease in SP1 levels mRNA compared with untreated tumors (Fig. 4C).

### Upregulation of miR‐24 expression increased radiosensitivity of tumor in mice bearing nasopharyngeal carcinoma tumor

**Figure 5:**
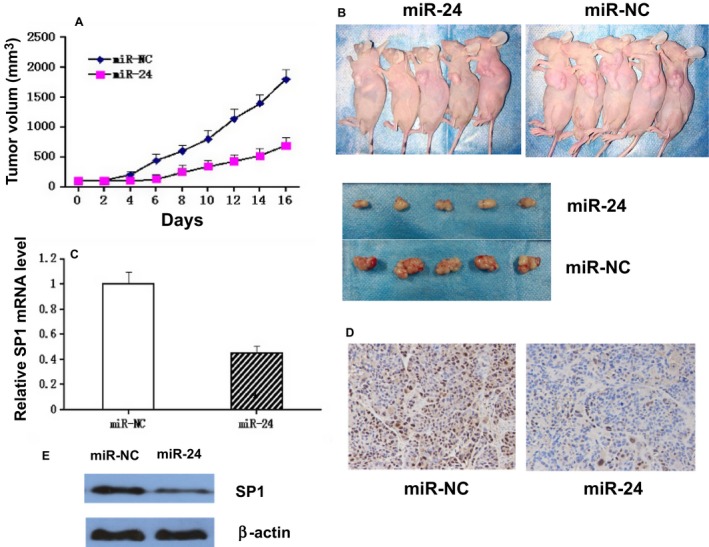
Effect of upregulated miR‐24 on tumor radiosensitivity and SP1 miRNA expression. Prior to injection into BALB/C mice, CNE1 cells were transfected with miR‐24 mimic. Tumors were irradiated with a single dose of 12 Gy/1f/1d. (A). Tumor growth in irradiated mice. (B). Photographs of tumors from miR‐24 and control groups. (C) Expression of SP1 was determined by using quantity RT‐PCR in tumors. The results were represented as mean ±SD. *P < 0.05 compared with negative control. (D). Immunohistochemical staining and quantification of expression of Sp1 was determined. The cytoplasm was counter‐stained with hematoxylin. (E). Western blot analysis of SP1 in nude mice xenograft tumor tissue.

To determine the effect of miR‐24 on the modulation of radiotherapy of tumors, CNE1 cells were transfected with miR‐24 mimic before subcutaneous transplantation in BALB/C mice. After local irradiation with a single dose of 12 Gy/1f/1d, the tumors' volumes were much lower than that of untreated tumors following irradiation (Figs. 5A and B). Additionally, compared with negative controls, upregulation of miR‐24 expression suppressed SP1 expression in tumors (Fig. 5C, D, and E).

## Discussion

miRNAs are known as key regulators of numerous cellular processes, and thus abnormal expression of miRNAs may be deemed closely related to the initiation and progression of malignant tumors 11, 12. In this study, we observed that miR‐24 is frequently downregulated in NPC cell lines and freshly frozen clinical specimens. Functional analyses revealed that depletion of miR‐24 suppressed NPC cell growth, proliferation and enhanced the radiosensitivity of NPC cells both in vitro and in vivo. Furthermore, SP1 was verified as a direct, functional target of miR‐24. Taken together, these data suggest that the identified miR‐24/Sp1 pathway contributes to the elucidation of the mechanisms of radiosensitivity in human NPC and that it may represent a potential target for therapy.

The main problem of treatment failure in many cancers is the resistance of tumor cells to irradiation. Recently, several microRNAs have been reported as playing an important role in regulating therapeutic efficacy in cancer therapy. High‐expression levels of miR‐214, miR‐200c, and miR‐199a have been found to induce therapeutic resistance in ovarian, esophageal, and cervical carcinomas, respectively 31, 32, 33. On the other hand, several microRNAs expressed at lower levels, such as let‐7i, miR‐181a, miR‐630, and miR‐128, were identified as closely related to therapeutic resistance in patients with ovarian, lung, and breast cancers, respectively 34, 35, 36, 37. It has recently been reported that several miRNAs, including miR‐608, and miR‐29c, are associated with the success of radiotherapy treatment, particularly in NPC 13, 14. In our recent study, cited above, miR‐24 was found to be significantly downregulated in NPC tissues and associated with poor prognosis. However, to date, it has only been reported that miR‐24 enhances the sensitivity of NPC cells in vitro 38. Thus far, the literature remains limited to studies of the biological function and molecular mechanism of miR‐24 in NPC. Therefore, we deemed it important to select miR‐24 for further investigation in this study.

miR‐24 is an abundant miRNA encoded by the corresponding gene that maps to human chromosome 9q22 and the 19p13 regions. It is well conserved between species and is expressed in normal tissues such as adipose, mammary gland, kidney, and differentiated skeletal muscle 15. It has been reported that miR‐24 acts as a tumor suppressor in a variety of human cancers, including tongue squamous cell carcinoma, osteosarcoma, bladder cancer, and gastric cancer 16, 17, 18, 19. According to this study, we confirmed firstly that miR‐24 was downregulated in NPC cell lines and freshly frozen tissues. We then conducted a series of biological experiments in order better to understand the function of miR‐24 in NPC. It was examined in vitro using the MTT assay and colony formation assay as well as in vivo using a xenograft tumor model to assess the effect of miR‐24 on NPC cell growth. Ectopic expression of miR‐24 significantly suppressed NPC cell proliferation and enhanced radiosensitivity both in vitro and in vivo. In a nutshell, these results demonstrate that the ability of miR‐24 to regulate cell growth and enhance radiosensitivity may contribute to the initiation and progression of NPC.

In order further to understand the mechanisms underlying the ability of miR‐24 to promote cell growth and enhance radiosensitivity in NPC, we identified SP1 as a potential target gene of miR‐24, based on bioinformatics analysis. Specificity protein 1 (SP1) protein is a member of the transcription factor family and is capable of binding GC/GT‐rich promoter elements through its C(2)H(2)‐type zinc fingers at C‐terminal domains. Through either stimulating or repressing the activity of gene promoters, the SP1 protein regulates expression of multiple genes involving in differentiation, cell cycle, and oncogenesis 39. A growing body of evidence indicates that the SP1 protein plays a critical role in many tumor types by regulating expression of the genes associated with growth and metastasis 40. For example, in lung cancer cells, overexpression of SP1 can upregulate the expression of ABCG2, which is one of the ATP binding cassette (ABC) transmembrane proteins that influence the chemotherapeutic resistance of cancer cells 41. However, there have been no studies focused on radioresistance of SP1 in nasopharyngeal cancer. In this study, a luciferase reporter gene assay verified SP1 as a direct target of miR‐24. In addition, the overexpression of miR‐24 significantly reduced the mRNA and protein expression of SP1. In our previous study, the expression of SP1 was upregulated in NPC cells and tissues, a result which is associated with poor prognosis and therapeutic resistance 42. This suggested that miR‐24 may suppress cell proliferation and enhance radiosensitivity of NPC cells by directly targeting SP1.

In conclusion, this study demonstrates that miR‐24 is frequently downregulated in NPC cell lines and freshly frozen clinical specimens. Functional analyses revealed that depletion of miR‐24 suppressed NPC cell growth and proliferation, while enhancing the radiosensitivity of NPC both in vitro and in vivo. Moreover, SP1 was verified as a direct, functional target of miR‐24. Taken together, these data suggest that the identified miR‐24/SPp1 pathway should help further our understanding of the mechanisms of radiosensitivity in human NPC and that it may represent a potential therapeutic target.

## Conflict of Interest

All authors declare that no financial competing interests exist.
